# Analysis of blood transfusion predictors in patients undergoing elective oesophagectomy for cancer

**DOI:** 10.1186/1471-2482-8-3

**Published:** 2008-01-25

**Authors:** Abraham A Ayantunde, Ming Y Ng, Saurov Pal, Neil T Welch, Simon L Parsons

**Affiliations:** 1 Department of Surgery, Nottingham City Hospital, Hucknall Road, Nottingham NG5 1PB, UK

## Abstract

**Background:**

Oesophagectomy for cancers is a major operation with significant blood loss and usage. Concerns exist about the side effects of blood transfusion, cost and availability of donated blood. We are not aware of any previous study that has evaluated predictive factors for perioperative blood transfusion in patients undergoing elective oesophagectomy for cancer.

This study aimed to audit the pattern of blood crossmatch and to evaluate factors predictive of transfusion requirements in oesophagectomy patients.

**Methods:**

Data was collected from the database of all patients who underwent oesophagectomy for cancer over a 2-year period. Clinico-pathological data collected included patients demographics, clinical factors, tumour histopathological data, preoperative and discharge haemoglobin levels, total blood loss, number of units of blood crossmatched pre-, intra- and postoperatively, number of blood units transfused, crossmatched units reused for another patient and number of blood units wasted.

Clinico-pathological variables were evaluated and logistic regression analysis was performed to determine which factors were predictive of blood transfusion.

**Results:**

A total of 145 patients with a male to female ratio of 2.5:1 and median age of 68 (40–85) years were audited. The mean preoperative haemoglobin (Hb) was 13.0 g/dl. 37% of males (Hb < 13.0 g/dl) and 29% of females (Hb < 11.5 g/dl) were anaemic preoperatively. A total of 1241 blood units were crossmatched and 316 units were transfused to 71 patients. Seventy four patients (51%) did not require blood transfusion during their hospital episode. 846 blood units not used for oesophagectomy patients were reused for other patients and 79 units were wasted. The overall crossmatch to transfusion ratio was 4:1 and reuse and wastage rates were 65.2% and 6.3% respectively. The independent predictors of blood transfusion include age >70 years, Hb level <11.0 g/dl, T-stage, presence of postoperative complications and anastomotic leak.

**Conclusion:**

The cohort of patients audited was over-crossmatched. The identified independent predictors of blood transfusion should be considered in preoperative blood ordering for oesophagectomy patients. This study has directly led to a reduction in the maximum surgical blood-ordering schedule for oesophagectomy to 2 units and a reaudit is underway.

## Background

Oesophageal cancer is the eighth leading cancer and the sixth leading cause of death from cancer worldwide. The prognosis for oesophageal cancer is poor and there has been an alarming rise in the incidence [1-3]. Anaemia is the most frequently observed haematological abnormality in cancer patients and occurs in about 30% of patients with oesophagogastric cancers (OGC) [4]. Our experience in Nottingham has shown that up to two-third of our patients presented with some degree of anaemia at the diagnosis of their cancer. We were able to demonstrate in a recent pilot study that more than half of newly diagnosed patients with gastric or oesophageal cancers were iron deficient [5].

Cancer-related anaemia is usually caused by multiple factors. The disease itself through blood loss and poor dietary intake due to dysphagia, anorexia and vomiting can cause anaemia in patients with cancer. Anaemia of chronic disease specific to cancer patients is believed to be due to activation of the immune and inflammatory pathways leading to release of cytokines [6,7]. It can also be exacerbated by side effects of treatment such as cytotoxic chemotherapy as a result of myelosuppression, suppression of erythropoietin production and/or a reduction in bone marrow response to erythropoietin [6]. Most patients with oesophagogastric cancers require operation to remove their cancer and this involves further blood loss. Sutton et al [8] have previously shown that oesophagectomy with extensive lymphadenectomy are usually accompanied by blood transfusion more commonly than radical operations for other gastrointestinal cancers. Anaemia can compound and delay recovery from such surgical procedure and the presence of anaemia prior to operation is associated with increase complication rates and transfusion requirements [9].

Blood transfusion is a rapid method of correcting anaemia and currently one of the common forms of treatment offered to cancer patients. However, concerns have been raised about the side effects of transfusion, cost and availability of donated blood [10]. The indications and trigger for blood transfusion have been redefined in the last decade so as to ensure that blood and blood products are considered and treated as medications in their own merit [11]. There is evidence that perioperative blood transfusion has immunomodulatory effect and adversely affects outcome in patients undergoing oesophagectomy for cancer [12]. We are not aware of any previous study that has evaluated predictive factors for perioperative blood transfusion in patients undergoing elective oesophagectomy for cancer.

This study aimed to audit the pattern of blood crossmatch and transfusion requirements in patients undergoing oesophagectomy for cancer. We evaluated the reuse and wastage rates of the blood primarily crossmatched for oesophagectomy patients and identified possible factors predictive of perioperative blood transfusion in our cohort of patients.

## Methods

Data was retrospectively collected from a database of all patients who underwent elective oesophagectomy for cancer at our institution between January 2003 and December 2004. The approval for this study was granted by the Nottingham City Hospital Audit and Clinical Governance Department. Operative procedures were carried out through a two-phase approach for oesophagectomy and a two-field lymphadenectomy. Clinico-pathological and laboratory data for these patients was obtained from our unit, the Hospital Information Support System (HISS), the blood bank databases and Patient Administration System (PAS). Clinico-pathological data collected included patients demographics, clinical factors, tumour histopathological data, preoperative and discharge haemoglobin levels, operative blood loss, number of units of blood crossmatched pre-, intra- and post-operatively, number of blood units transfused, crossmatched units reused for another patient and number of blood units wasted.

This audit covered the period when the recommended routine preoperative crossmatching by our Oesophagogastric unit for oesophagectomy was 4–6 units. Anaemia was defined on the basis of haemoglobin levels below the lower limit of normal for our laboratory reference values in accordance with the World Health Organization (WHO) criteria [13] with Hb < 13.0 g/dl in males and <11.5 g/dl in females. Total blood loss was calculated as the sum of intraoperative blood measured by the anaesthesiologist according to the contents of the suction bottles & the weight change of surgical swabs and postoperative blood loss. The criteria used as guidelines for blood transfusion in our unit during the audited period included significant blood loss >800 ml, haemodynamic instability or persistent postoperative haemoglobin <8.0 g/dl. The total blood loss and transfusion requirements were compared between anaemic and non-anaemic patient groups. We then evaluated the effect of various clinico-pathological factors on blood transfusion requirements.

### Statistical analysis

Statistical analysis was performed using the SPSS version 13.0 for windows-software (SPSS, Chicago, Illinois, USA) to present descriptive statistics. The mean and median values were calculated for continuous and discrete variables respectively. Univariate and multivariate analyses were performed to determine the risk factors for blood transfusion. Statistical significance was tested using Mann-Whitney U-test or student t-test where appropriate. Variables that demonstrated a significant relationship on univariate analysis were included in a multiple stepwise logistic regression analysis to identify the significant independent predictors of blood transfusion. The level of significance was set at p-value less than 0.05.

## Results

A total of 145 consecutive patients were included in the study with 104 males and 41 females (Male: Female ratio of 2.5:1). The median age was 68 (40–85) years. The predominant histological type was adenocarcinoma (107) compared with squamous cell carcinoma (38). The mean pre-operative haemoglobin (Hb) was 13.0 (9–17.5) g/dl. Of the male group, 39 patients (37.5%) were anaemic preoperatively (Hb < 13.0 g/dl) and 12 patients (29.7%) of the female group were anaemic preoperatively (Hb < 11.5 g/dl). There was no difference in the distribution of co-morbid factors between the anaemic and non-anaemic groups. The mean discharge Hb was 11.0 (7.9 – 15.6) g/dl. The median operative blood loss was 700 ml (150 – 2400) with a mean of 849 ml and there was no significant difference between the anaemic and non-anaemic patients (p = 0.343). Forty-four percent of the patients had neoadjuvant chemotherapy and there was no significant difference in operative blood loss between this group and those who did not receive neoadjuvant chemotherapy.

A total of 1241 blood units were crossmatched and 74% were performed preoperatively, 3% between intra-operative period to 48 hours postoperatively and 23% from 48 hours postoperatively to discharge. The median number of blood units crossmatched from the preoperative period to discharge was 6 (0 – 34) units with a mean of 8.6 units. The median number of blood units crossmatched preoperatively was 6 (0 – 23) units with a mean of 6.3 units. The median number of blood units crossmatched from the start of the operation to 48 hours postoperatively was 0 (0 – 7) units with a mean of 0.3 unit. The median number of blood units crossmatched from 48 hours postoperatively to discharge was 0 (0 – 30) units with a mean of 2.0 units. Thirty-nine percent of the patients had multiple peri-operative blood crossmatch over the upper limit of 6 units that was routinely performed during the audited period and the reasons are outlined in Table 1.

**Table 1:** Reasons for multiple blood ordering of more than six units in 56 patients

1. Severe anaemia requiring preoperative top up transfusion
2. Operation cancellation
(i) Medical reasons
(ii) Unavailability of intensive care unit bed
3. Excessive intraoperative bleeding
4. Reoperation
(i) Ischaemic anastomotic site or bowel
(ii) Postoperative bleeding
(iii) Chyle leak
(iv) Massive gastrointestinal bleeding
(v) Empyema
5. Postoperative complications
(i) Anastomotic leak
(ii) Postoperative bleeding
(iii) Sepsis

**Figure 1:**
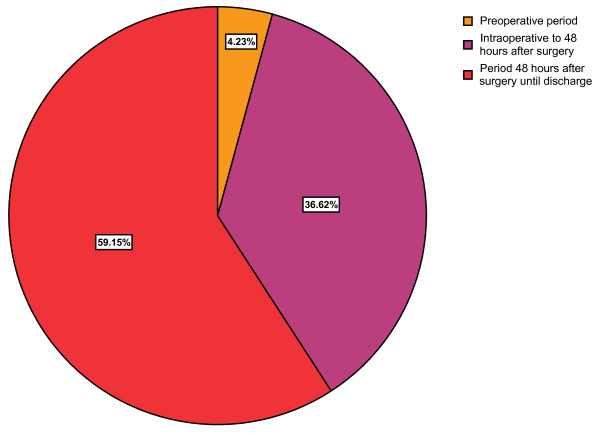
Pie chart showing the time period of perioperative blood transfusion in patients undergoing oesophagectomy.

Forty-nine percent (71/145) of the patients were transfused with a total of 316 blood units. Ninety-six percent of the transfusions occurred between intraoperative period and discharge (Figure 1). The median number of blood transfused was 4 (1 – 18) units with a mean of 4.5 units in this group of patients. Median operative blood loss was higher in patients who were transfused than those that were not transfused but the difference did not reach a statistical significance (p-value = 0.168). The risk factors for perioperative blood transfusion on univariate analysis are shown in Table 2. The independent predictors of blood transfusion are age more than 70 years, Hb level less than 11.0 g/dl, T-stage, presence of perioperative complications and anastomotic leak (Table 3). There was no difference between the two groups in relation to gender, tumour location, histology, differentiation, and lymph node involvement. A direct correlation between the severity of preoperative anaemia and perioperative blood transfusion requirements was observed in spite of similar operative blood loss in the two groups (p = 0.013).

**Table 2:** Risk factors for perioperative blood transfusion in 145 patients undergoing oesophagectomy

**Factors**	**Transfused (71)**	**Not transfused (74)**	**P-value**
Sex:			0.148
Male	47	57	
Female	24	17	
Age:			0.0001
<70 years	31	54	
>70 years	40	20	
Comorbid factors:			0.002
Yes	45	28	
No	26	46	
ASA Class:			0.004
1	8	18	
2	44	48	
3	19	8	
ECOG score:			0.005
0	27	45	
1	31	23	
2	10	4	
3	3	2	
Haemoglobin levels:			0.001
<11.0 g/dl	15	2	
≥ 11.0 g/dl	56	72	
Tumour site:			0.836
Upper third	1	1	
Middle third	8	6	
Lower third	18	16	
GOJ	44	51	
Neoadjuvant chemotherapy:			0.266
Yes	28	36	
No	43	38	
Tumour histology:			0.200
SCC	22	16	
ADC	49	58	
Tumour grade:			0.870
Well	12	13	
Moderately	26	25	
Poorly	33	36	
T-stage:			0.002
1	5	16	
2	9	16	
3	55	42	
4	2	0	
N-stage:			0.166
N0	34	27	
N1	37	47	
Operative blood loss:			0.715
<800 ml	43	47	
>800 ml	28	27	
Postoperative complications:			0.0001
Yes	46	25	
No	25	49	
Anastomotic leak:			0.001
Yes	13	1	
No	58	73	

ECOG, Eastern Cooperative Oncology Group; GOJ, Gastro-oesophageal junction;

SCC, Squamous cell carcinoma; ADC, Adenocarcinoma

**Table 3:** Independent predictors of blood transfusion by logistic regression analysis

**Factor**	**p-value**	**RR**	**95% Confidence Interval**
Age >70 years	0.044	2.328	1.022 – 5.304
Hb level <11.0 g/dl	0.0001	3.225	1.797 – 5.786
T-stage	0.004	2.436	1.249 – 5.762
Perioperative complications	0.015	2.801	1.219 – 6.436
Anastomotic leak	0.028	14.545	1.331 – 158.903

RR, Relative risk

A total of 846 blood units that were crossmatched primarily for oesophagectomy patients were reused for another group of patients and 79 blood units were wasted overall. The overall crossmatch to transfusion ratio (C/T ratio) was 4:1 with overall reuse and wastage rates of 65.2% and 6.3% respectively. C/T ratio was significantly higher in patients who had six or less number of blood units crossmatched compared with patients with more than six units crossmatched (C/T ratio 8.5:1 versus 2.8:1; p = 0.001). The wastage rate was twice as high in the former compared with the latter patient groups (9% versus 4.5%).

## Discussion

Surgery is still the main treatment option for oesophageal cancer and recent advances in surgical and anaesthetic techniques with improvements in postoperative care have reduced the risks of oesophageal resection to an acceptable level. It has been reported that oesophagectomy with extensive lymphadenectomy is usually accompanied by blood transfusion more commonly than radical operations for other gastrointestinal cancers [8,12]. It is therefore customary that patients planned for oesophagectomy for cancer are routinely grouped and crossmatched so as to ensure that blood is readily available during and immediately after the operation. There have been several studies in the last three decades reviewing blood ordering and transfusion practices on account of gross over ordering of blood much in excess of actual or anticipated needs [14-17]. These studies resulted from increasing demand for blood and blood products together with rising costs and increased public and medical concerns regarding transfusion-associated infections and immunosuppression.

Surgical indications have been shown to account for more than half of red cells blood transfusion in the United States [18]. However, it is known that many units of blood routinely ordered for surgery are never utilised, resulting in extra workload for laboratory staff and expenses for the blood banks [15-17]. Such blood units are held in reserve and therefore not immediately available for other patients, which can lead to loss of shelf life and eventual wastage. The cost of blood transfusions to the National Health Service (NHS) in 2000/2001 was estimated to be £898 millions, representing a 256% increase since 1994/1995 in the UK [19]. The demand for blood and blood products has been predicted to increase by 4.9% by 2008 [20]. The increasing demand for blood, increase in ageing population, possible fall in the donor pool and introduction of further screening tests for donated blood will lead to scarcity and further increase in the costs of blood transfusion [19,20].

This is the first study to the best of our knowledge that evaluated predictive clinico-pathological factors for blood transfusion in patients undergoing elective oesophagectomy for cancer. Four to six units of blood were routinely crossmatched preoperatively for patients undergoing oesophagectomy for cancer at the time covered by this audit in our unit. Significant number of our patients had preoperative anaemia. Our median operative blood loss of 700 ml and transfusion rate are comparable to those reported in the literature [12]. There was no statistically significant difference between the operative blood loss between the anaemic and non-anaemic patients. Patients who were older than 70 years had significantly more tendency for perioperative blood transfusion and we believe this may be related to the presence of more co-morbid factors in them than the younger patients. The younger patients understandably may also have more appropriate physiological compensatory mechanisms to blood loss than the older group.

Our results showed that the number of blood units crossmatched significantly exceeded that which was actually transfused to the patients. Forty-nine percent of our patients were transfused with a mean of 4.5 units and only a third of these patients had more than five units of blood transfused. This finding supports previous reports pointing to the tendency to over crossmatching of blood for patients undergoing major operations [15-17]. The British Committee for Standards in Haematology (BCSH) formulated guidelines stating that, in general, the ratio of the number of blood units ordered and transfused should not normally exceed 2:1 [21]. The overall crossmacth to transfusion ratio was twice the recommended value by the BCSH and in fact fifty-two percent of the transfused patients in this study had blood crossmatched to transfusion ratio above 2:1. This evidently showed that the cohort of patients audited was over-crossmatched and this study has directly led to a reduction in the maximum surgical blood ordering schedule (MSBOS) for oesophagectomy to 2 units. Palmer et al [17] have previously reported that a patient-specific blood ordering system (PSBOS) is more accurate in predicting potential perioperative blood transfusion. Sixty-eight percent of the unused blood primarily crossmatched for our oesophagectomy patients were subsequently reused for other patients but still, 6.3% of blood units were wasted. This wastage rate falls within the reported rates by previous authors [22,23]. A significant proportion of the wastage in this study was due to expiration of the shelf life of blood units.

The adoption of PSBOS approach has the potential and capacity to reduce the workload in the transfusion laboratories, reduce cost, conserve already scarce blood resources and prevent unnecessary blood transfusion and wastage. The rational way to minimize blood wastage and reduce the crossmatch to transfusion ratio is by more precisely predicting and estimating an individual patient's likelihood of requiring transfusion perioperatively. We have evaluated and identified some independent factors that are predictive of patients' likelihood of blood transfusion requirements such as age above 70 years, preoperative haemoglobin less than 11.0 g/dl, locally advanced tumour, the presence of perioperative complications especially postoperative sepsis and anastomotic leak. The results of this audit support other previous studies [15,16,21,24,25]. The current study demonstrated no relationship between the need for blood transfusion and gender, tumour site, histology, differentiation, nodal involvement and the use of neoadjuvant chemotherapy.

The local Hospital Transfusion Committee (HTC) may be able to monitor the effectiveness of the blood requesting policy using the crossmatch to transfusion ratio. Preoperative strategies should incorporate blood ordering services aimed at reducing over crossmatch and avoid wastage of a scarce resource. Greater precision in crossmatch to transfusion ratio can be further improved by careful estimation of an individual patient's likelihood of having blood transfusion perioperatively through the application of identified clinico-pathological and operative risk factors. This approach is likely to address the issues of demand versus supply under the current circumstances of concerns relating to the costs, safety of blood and blood products. Improvements and advances in surgical and anaesthetic techniques with better perioperative care may therefore mean that fewer patients are requiring blood transfusion following their operation.

Thirty-nine percent of our patients had multiple perioperative blood crossmatch whereby there were several instances when blood sample was sent for one or more units of blood to be crossmatched. The reasons for this practice included operation cancellations, severe preoperative anaemia necessitating transfusion before surgery and perioperative complications. Complications leading to multiple blood crossmatch and transfusions were anastomotic leakage, sepsis, reoperation for various reasons and postoperative bleeding. The crossmatch to transfusion ratio was significantly lower (C/T ratio 2.8:1 versus 8.5:1) and wastage rates was half (4.5% versus 9%) in patients who had multiple crossmatch compared with those with six or less units of blood crossmatched. We do agree with Palmer et al [17] that an individual patient-specific blood ordering system may be more accurate in reducing unnecessary crossmatch than the maximal surgical blood ordering system. PSBOS can only be effectively applied by predicting the likelihood of an individual patient risk of receiving blood transfusion in the course of their hospital episode for elective operations. Varney and Guest [19] in an economic study of the annual cost of blood transfusions in the UK for 2000/2001 estimated that the average cost of an adult transfusion of a unit of red blood cells to the NHS was £635. The estimated cost of a unit of red blood cells including the laboratory services in our hospital is £132 and this, when calculated for 79 units of blood wasted in this audit will translate into a total of £10,428.

## Conclusion

The cohort of patients audited in this study was over-crossmatched. The future trends are likely to be related to major changes in the blood ordering and transfusion practice as a result of effect of demand versus supply, evolving surgical and anaesthetic techniques, redefinition of more objective transfusion triggers, availability of possibly cheaper alternatives to blood and blood products and the public's and clinicians' perceptions of safety of blood transfusion. The rising costs of blood transfusion and potential reduction in donor pool make it imperative for us to continue to utilise the scarce blood resources more effectively. The identified independent predictors of blood transfusion should be considered in preoperative blood ordering for patients undergoing oesophagectomy.

## Abbreviations

ADC, Adenocarcinoma; BCSH, British Committee for Standards in Haematology; C/T ratio, Crossmatch to transfusion ratio; ECOG, Eastern Cooperative Oncology Group; GOJ, Gastro-oesopjageal junction; Hb, Haemoglobin; HISS, Hospital Information Support System; HTC, Hospital Transfusion Committee; MSBOS, Maximum Surgical Blood Ordering Schedule; NHS, National Health Service; OGC, Oesophagogastric cancer; PAS, Patient Administration System; PSBOS, Patient-Specific Blood Ordering System; SCC, Squamous cell carcinoma; SPSS, Statistical Package for Social Science; WHO, World Health Organization;

## Competing interests

The author(s) declare that they have no competing interests.

## Authors' contributions

AAA participated in the conception, design, acquisition of the data, statistical analysis of the data, interpretation of the data, drafting of the manuscript, critical revision of the manuscript and supervision.

MYN participated in the conception, design, acquisition of the data, interpretation of the data and critical revision of the manuscript.

SP participated in the conception, design, acquisition of the data interpretation of the data and critical revision of the manuscript.

NTW participated in the conception, design, acquisition of the data, statistical analysis of the data, interpretation of the data, drafting of the manuscript, critical revision of the manuscript and supervision.

SLP participated in the conception, design, acquisition of the data, statistical analysis of the data, interpretation of the data, drafting of the manuscript, critical revision of the manuscript and supervision.

All authors read and approved the final manuscript.

## Pre-publication history

The pre-publication history for this paper can be accessed here:


